# Theory of Response to Perturbations in Non-Hermitian Systems Using Five-Hilbert-Space Reformulation of Unitary Quantum Mechanics

**DOI:** 10.3390/e22010080

**Published:** 2020-01-09

**Authors:** Miloslav Znojil

**Affiliations:** 1 Institute of System Science, Durban University of Technology, P. O. Box 1334, Durban 4000, South Africa; znojil@ujf.cas.cz; 2 The Czech Academy of Sciences, Nuclear Physics Institute, Hlavní 130, 250 68 Řež, Czech Republic

**Keywords:** hidden Hermiticity, Hilbert space metric, size of perturbations, stability, PT symmetry, unitary quantum evolution

## Abstract

Non-Hermitian quantum-Hamiltonian-candidate combination $H_{\lambda }$ of a non-Hermitian unperturbed operator $H=H_{0}$ with an arbitrary “small” non-Hermitian perturbation λW is given a mathematically consistent unitary-evolution interpretation. The formalism generalizes the conventional constructive Rayleigh–Schrödinger perturbation expansion technique. It is sufficiently general to take into account the well known formal ambiguity of reconstruction of the correct physical Hilbert space of states. The possibility of removal of the ambiguity via a complete, irreducible set of observables is also discussed.

## 1. Introduction

“How much would a small change of a Hamiltonian influence the measurable properties of a unitary quantum system?” This is the question answered, in general terms, by perturbation theory [1]. In a less abstract setting, it makes sense to distinguish between two alternative scenarios, viz., Hermitian theory and non-Hermitian theory. In our present paper, we intend to study a few less trivial questions emerging in the latter context.

We feel motivated by observation that conventional textbooks on quantum mechanics rarely leave the former, Hermitian-theory framework [2]. For our present purposes, we will call this framework the “single Hilbert space (1HS) formulation of quantum mechanics” because, typically, the states are represented in a fixed, preselected Hilbert space (say, L). In the so-called Schrödinger picture, the evolution of wave functions (denoted by curly-ket symbol |•≻ in what follows) is then required controlled by a suitable self-adjoint Hamiltonian (say, $h=h^{†}$), i.e., by Schrödinger equation

(1) $$i\frac{d}{dt}|\psi \left(t\right)\;\;≻\;\;=\;h\;|\psi \left(t\right)\;\;≻\;\;\;\;\;\;\;|\psi \left(t\right)\;\;≻\;\;\in \;L\;.$$

In stationary models the construction then degenerates to diagonalization,

(2) $$h\;|\psi^{\left(n\right)}\;\;≻\;=E^{\left(n\right)}\;\left|\psi^{\left(n\right)}\;\;≻\;\;\;\;\;\;\right|\psi^{\left(n\right)}\;\;≻\;\;\in \;L\;\;\;\;\;\;\;\;n=0,1,\ldots \;.$$

The study of influence of perturbations is then performed in the same 1HS framework. Reference is made to the well known Stone theorem [3]. The self-adjointness is postulated also for perturbations,

(3) $$h\;\to \;h_{\lambda }=h+\lambda v=h_{\lambda }^{†}\;.$$

Any non-Hermiticity is excluded as forbidden. The estimates of energies $E_{\lambda }^{\left(n\right)}$ and/or of state vectors $|\psi_{\lambda }^{\left(n\right)}\;\;≻\;\;\in \;L$ are calculated using, e.g., the Rayleigh–Schrödinger power-series ansatzs [2].

By Bender with Boettcher [4], the apparently obvious constraint of self-adjointness (3) has been claimed over-restrictive. In Section 2 and Section 3, the subsequent consistent amendment of the formalism will be reviewed and summarized (cf. also the older references [5,6] and newer reviews [7,8,9,10] in this respect). We will summarize the current state of the art. Its abstract outline will be complemented by a schematic, “relativistic” illustrative example.

It is worth adding that, in the current literature, such a version of the unitary quantum theory became widely known under the most popular names of the “non-Hermitian but PT- symmetric” [7] *alias* “pseudo-Hermitian” [8] quantum mechanics. More explanatory than “non-Hermitian” would be, certainly, “quasi-Hermitian” [6,8] or “crypto-Hermitian” [11]. A few other comments on the not yet unified terminology may be also found in our papers [12,13]. Here, in a way proposed in [14], the corresponding innovative but still strictly unitary amendment of Schrödinger picture will be simply called the “three Hilbert space (3HS) formulation of quantum mechanics”.

In this framework, our key methodical observation is that the underlying mathematics is comparatively well understood only under a tacit assumption that there are no perturbations which could enter the game. In this situation, the quantum physicists communicating with experimentalists and working with the non-Hermitian Hamiltonians with real spectra have two options, viz., the closed-system option, and the open-system option. In the latter case, one admits that the corresponding Schrödinger equation describes an open, unstable quantum system (such an option and physical interpretation of non-Hermiticity are often found advocated also by mathematicians: see, e.g., Refs. [15,16,17,18]).

In the former case (this option is to be chosen and defended in what follows), the physicists insist on the unitarity of evolution and on the hiddenly Hermitian nature of Hamiltonians. Under these assumptions, one encounters the necessity of working, in the case of any parameter-dependent crypto-Hermitian Hamiltonian $H_{\lambda }$, with a multiplet of parallel, λ-numbered versions of the 3HS formalism of course.

This is a paradox. A clarification of this paradox is one of the main aims of our present paper. It will be formulated in Section 4. In Section 4.1, we will explain that, in the perturbed case, it is sufficient to keep the parameter fixed, dealing just with two (viz., λ≠0 and λ=0) separate versions of the 3HS formalism. In Section 4.2, we describe the merger of the two pictures yielding the ultimate “five Hilbert space (5HS) formulation of quantum mechanics”.

The amended and simplified final version of our general non-Hermitian perturbation theory is described in Section 5. It is characterized by a reduction of the doublet of the two (viz., λ≠0 and λ=0) “friendliest” Hilbert spaces to the single one, with λ=0. In this space (denoted by the dedicated symbol K), the set of the “relevant” perturbations of *H* is finally introduced as the most natural form of the dynamical-input information about the system.

A few related technical results are then developed and discussed in Section 6. Emphasis is put upon a simplification of the scheme based on a formal return to a single auxiliary Hilbert space, and upon the study of properties of the new, “effective” physical metric. A few explicit aspects of the construction of the perturbation-expansion corrections are outlined, and their recurrent nature is underlined. In the resulting analogue and extension of the standard Rayleigh–Schrödinger perturbation-expansion recipe, the number of the relevant Hilbert spaces will be found reduced back to three again.

In Section 7, we will discuss a few practical aspects and consequences of the formalism, re-emphasizing that, after the innovation, multiple phenomenological merits provided by the conventional perturbative model-building strategies still remain unchanged. In particular, we add a few more comments on the difference between the open and closed quantum systems (cf. Section 7.1) or on the problem of stability (cf. Section 7.2), etc.

In the last Section 8, our present upgrade of the perturbation expansion techniques will be summarized.

## 2. Non-Hermitian Hamiltonians and Unitarity in Disguise

Due to the Dyson’s study of ferromagnetism [5], due to the Čížek’s coupled-cluster calculations in quantum chemistry [19], and due to the successful variational interacting-boson-model evaluations of energy levels in heavy nuclei [20], it has widely been accepted that at least some of the most critical technical obstacles emerging in multiple nontrivial calculations can be circumvented when one weakens, in a suitable manner, the Hermiticity assumption. A way towards such an innovation has been found to lie in a transfer of Equations (1) and (2) from the (by assumption, overcomplicated) “inaccessible” textbook Hilbert space L to its unitary non-equivalent Hilbert-space alternative (say, K). Dyson recommended to pick up a suitable invertible operator Ω carrying, typically, information about correlations, and leading to a “prediagonalization” of a given realistic but overcomplicated Hamiltonian $h=h^{†}$,

(4) $$h=\Omega \;H\;\Omega^{-1}\;\;\;\;\;\;\Omega^{†}\Omega =\Theta \neq I\;.$$

In the formally equivalent time-dependent Schrödinger equation

(5) $$i\frac{d}{dt}|\psi \left(t\right)\rangle \;=\;H\;|\psi \left(t\right)\rangle \;\;\;\;\;\;|\psi \left(t\right)\rangle \;\in \;K\;\;\;\;\;H\neq H^{†}\;$$

as well as in its time-independent complement

(6) $$H\;|\psi^{\left(n\right)}\rangle =E^{\left(n\right)}\;|\psi^{\left(n\right)}\rangle \;\;\;\;\;|\psi^{\left(n\right)}\rangle \;\in \;K\;\;\;\;\;\;\;\;n=0,1,\ldots \;$$

such a transformation (also known, in numerical mathematics, as “preconditioning”) can only be considered meaningful if it leads to a truly perceivable simplification of the diagonalization.

The new, isospectral form *H* of the Hamiltonian must prove to be user-friendlier. In the most successful implementations, the optimal, Dyson-recommended non-unitary choice of mapping Ω is essential. The methodical gain provided by prediagonalization h→H is accompanied by the loss represented by the emergence of the non-Hermiticity of *H*. The self-adjointness of $h=h^{†}$ in L gets replaced by the more complicated quasi-Hermiticity relation in K,

(7) $$H^{†}\Theta =\Theta \;H\;.$$

Operator $\Theta =\Omega^{†}\Omega$ is usually called physical Hilbert-space metric [6].

The trade-off has fiercely been criticized by Dieudonné [21] (cf. also the most recent warnings in [15,22,23]). Only the acceptance of a few additional restrictive technical assumptions made the recipe mathematically satisfactory and consistent (for example, the strongly counterintuitive boundedness of all of the operators of observables was required in [6]).

Some of the latter technical constraints may be given a natural form when one re-qualifies operator Ω as a map which connects the space L of textbooks with another, auxiliary Hilbert space K. This proves useful, e.g., in the so-called interacting boson models of nuclei where the antisymmetry of wave functions imposed in the fermionic Fock space L is replaced by the boson-space statistics in K [20]. This enables us to write

(8) |ψ≻=Ω|ψ〉|ψ≻∈L|ψ〉∈K.

Such a notation convention simplifies the necessary change of the Hilbert space, viz., K→H. This move completing the 3HS scheme is easy because one merely amends the inner product,

(9) $${\langle \psi_{a}|\psi_{b}\rangle }_{\left(H\right)}=\langle \psi_{a}\left|\Theta \right|\psi_{b}\rangle_{\left(K\right)}\;.$$

The two Hilbert spaces L and H now become, from the point of view of making experimental predictions, equivalent (cf., e.g., reviews [6,8,24] for more details). Thus, the Dyson-inspired constructive 3HS recipe acquires the following three-step structure:

(10) inthefirststep,aunitaryquantumsystemwithaconventionalHamiltonianh=h†definedinatextbookHilbertspaceLisfoundprohibitivelycomplicated;itisdiscarded;simplificationsaresought;action:preconditioning↙↖↘comparison:thesamephysicsinthesecondstep,calculationsaremadefeasibleusinganauxiliaryHilbertspaceKandisospectralH=Ω−1hΩ≠H†(simplificationisachieved);⟶rehermitizationinthelaststep,thesameHismadeselfadjointviainnerproductmetricΘ=Ω†Ω≠IyieldingthestandardHilbertspaceH(predictionsarerenderedpossible).

The construction proceeds from a realistic self-adjoint h and from a preselected Ω to a friendlier *H* which is Hermitian in H,

(11) $$H=\Theta^{-1}H^{†}\Theta :=H^{‡}.$$

This relation demonstrates the unitary equivalence between two alternative physical Hilbert spaces H and L. In K itself, the upper-case Hamiltonians with property (7) are non-Hermitian. This means that the numerical diagonalization algorithms may be less efficient [25].

## 3. Inverted Unperturbed Flowchart

### 3.1. The Necessity of the Reconstruction of Metric

Once we decide to start, directly, from a suitable *H* in K, we have to follow the recipes by Scholtz et al. [6], or by Bender with coauthors [7]. The necessary process of the Hermitization K→H has to involve not only *H* but also, possibly, the knowledge of a quasi-parity Q [26], of a charge C [7], or of any other “compatible” [27] candidate Λ for an observable [6]. The ultimate aim of the calculations then becomes the reconstruction of metric Θ(H) [28]. Naturally, the formalism still can provide all of the relevant experimental predictions as well as a consistent picture of physical reality, in principle at least. In practice, the evaluation of predictions will be guided by the following inverted triangular diagram:

(12) step(A):onepicksupanunphysicalbutuser-friendlyHilbertspaceKandanon—HermitianHwithrealspectrum,i.e.,abonafideHamiltonian;⟶hermitizationstep(B):oneconstructsaneligiblemetricΘ=Ω†Ω(s.t.H†Θ=ΘH),i.e.,physicalHilbertspaceHinwhichtheevolutionbecomesunitary;↗↙equivalentpredictionsstep(C):foraclearerphysicalinterpretationonemayreconstructalsotheconventionalHamiltonianh=ΩHΩ−1=h†inthetextbookHilbertspaceL.

This characterizes the theoretical construction flowchart used, in particular, when one speaks about the “non-Hermitian but PT—symmetric” Hamiltonians *H* [7], or about the P—pseudo-Hermitian Hamiltonians *H* [8], etc. An explicit two-by-two matrix illustration of the backward assignment H→Θ(H) will be provided in the following subsection.

### 3.2. The Ambiguity of the Reconstruction of Metric

Operator Θ is, in general, non-universal and Hamiltonian-dependent. Its use is easily shown to convert the conventional Hermiticity $h=h^{†}$ of Hamiltonian (4) in L into the formally equivalent quasi-Hermiticity property

(13) $$H=\Theta^{-1}H^{†}\Theta \;$$

of its non-Hermitian preconditioned isospectral partner in K.

A suitable elementary but still sufficiently realistic 2N by 2N-matrix example of the formalism may be found, e.g., in Ref. [29]. The physical contents of the theory has been illustrated there, in the context of relativistic quantum mechanics, using the discrete version of the free Klein–Gordon Hamiltonian $H^{\left(KG\right)}$. Even the first nontrivial special case of this model, with N=1, offers a nontrivial but exactly solvable two-level quantum system characterized by the real two-by-two-matrix Hamiltonian

(14) $$H=H^{\left(KG\right)}\left(\tau \right)=\left[\begin{array}{cc} 0 & \exp2\tau \\ 1 & 0 \end{array}\right]\neq H^{†}\;.$$

The dynamics are controlled here by a single real parameter τ∈(−∞,∞). The toy-model Hamiltonian (14) is PT—symmetric, with antilinear T representing Hermitian conjugation, and with the Pauli-matrix parity $P=\sigma_{x}$. In spite of the manifest non-Hermiticity of $H^{\left(KG\right)}\left(\tau \right)$ at τ≠0, the bound-state energies are real and non-degenerate, $E=E_{\pm }=\pm \exp\tau$. The model is suitable for illustrative purposes because the list of its properties survives a transition to the discrete free Klein–Gordon Hamiltonians with arbitrary matrix dimensions 2N (cf. [30]).

Every element $H^{\left(KG\right)}$ of the latter family is non-Hermitian, but it still has been shown to admit the conventional probabilistic interpretation. This goal is achieved in the third Hilbert space H in which, by construction, the eigenstates of the upper-case Hamiltonian *H* really do acquire their conventional probabilistic physical interpretation of wave functions. In comparison, the role of the friendlier Hilbert space K (with non-amended inner product) remains just that of a mathematical playground.

The construction of the correct physical inner product (i.e., of the non-equivalent space H) is a key to the phenomenological acceptability of the model. For illustrative Equation (14), the space $K=K^{\left(KG\right)}$ is the real two-dimensional vector space. In such a model, it is easy to show that the Hamiltonian $H^{\left(KG\right)}$ will satisfy the “hidden”, H—space-Hermiticity relation (13) if and only if the following, entirely general amended form

(15) $$\Theta =\Theta^{\left(KG\right)}\left(\tau ,\beta \right)=\left[\begin{array}{cc} \exp(-\tau ) & \beta \\ \beta & \exp\tau \end{array}\right]=\Theta^{†}\;$$

of the metric is used upgrading $K^{\left(KG\right)}$ into our ultimate physical Hilbert space $H^{\left(KG\right)}$.

Matrix (15) contains also another, independent real variable β which must keep it positive definite [6], i.e., which must be such that |β|<1. Its variability reflects the generic ambiguity of the assignment $H^{\left(KG\right)}\to \Theta^{\left(KG\right)}\to H^{\left(KG\right)}$. The free variability of such a “redundant” parameter must be kept in mind as an inseparable, characteristic part of the 3HS formulation of quantum theory [31].

According to review paper [6], there exists the possibility of a systematic, physics-based removal of the ambiguity mediated by the completion of the set of the so-called irreducible observables (say, $\Lambda_{j}$, $j=1,2,\ldots ,j_{\max}$). In our most elementary real-matrix model (14) leading to the single-parameter ambiguity in metric (15), for example, one would need just $j_{\max}=1$, i.e., just one “missing” real-matrix candidate for observable

(16) $$\Lambda =\left[\begin{array}{cc} a & b\\ c & d \end{array}\right]\;.$$

As long as its observability property $\Lambda^{†}\Theta^{\left(KG\right)}\left(\tau ,\beta \right)=\Theta^{\left(KG\right)}\left(\tau ,\beta \right)\Lambda$ forms the set of four linear algebraic equations, we have to distinguish between the solutions with d=a and d≠a. As long as the former case (of which the Hamiltonian itself is the special case with a=0) does not determine the value of β, all of the eligible candidates Λ belong to the latter case. For all of them, our set of four equations has the solution

(17) $$\beta =\beta \left(a,b,c,d,\tau \right)=\frac{c\;\exp(\tau )-b\;\exp(-\tau )}{d-a}.\;$$

Thus, we proved the following elementary but instructive result.

**Lemma** **1.** *Once we guarantee that the four eligible real parameters in the “additional dynamical input” observable (16) are such that |β(a,b,c,d,τ)|<1 in Equation (17), the corresponding correct physical Hilbert-space metric (15) becomes unique.*

## 4. Five-Hilbert-Space Formulation of Quantum Mechanics

The three-Hilbert-space (3HS) formulation of quantum mechanics as reviewed in the preceding section offers an explanation why, under certain general mathematical assumptions, any given parameter-independent quasi-Hermitian Hamiltonian *H* with real spectrum can play the role of the generator of quantum evolution which is unitary. In essence, the explanation is based on the clarification that only the two Hilbert spaces in question (viz., L and H) are physical (i.e., unitary equivalent) while the third Hilbert space K (in which calculations are being performed) yields unphysical values of inner products.

### 4.1. Parameter-Dependent 3HS Theory

In the overall 3HS framework the correct physical interpretation of a quantum system in question becomes much more complicated when the upper-case Hamiltonians are allowed to vary with a parameter [27]. Even the inspection of our most elementary τ—dependent toy model (14) reveals that the change of the parameter may modify the physical Hilbert-space metric. Moreover, even at a fixed τ, the specification of the metric remains ambiguous—notice that its form (15) varies with another free parameter β. After a change of τ, our choice of β may be different, β=β(τ).

For an arbitrary crypto-Hermitian 3HS Hamiltonian (or, if needed, for the operators of arbitrary other observable quantities), both of the latter remarks are truly essential. In particular, they are essential for an appropriate and consistent introduction of the concept of perturbations of the Hamiltonians in Schrödinger picture [32]. Indeed, any deviation from the “unperturbed” scenario, even with a “very small” parameter λ in

(18) $$H\;\to \;H_{\lambda }=H+O\left(\lambda \right)\neq H_{\lambda }^{†}\;$$

makes it necessary to reopen the question of the applicability of the conventional Rayleigh–Schrödinger power series in λ.

Our present main task is to demonstrate why the perturbed forms (18) of the 3HS-related non-Hermitian upper-case Hamiltonians really require a deep change of the conventional perturbation-approximation approaches. We will show under which conditions these deeply anomalous models can still be made a part of a consistent quantum theory of unitary evolution.

Let us start by recalling that whenever one alters the Hamiltonian (say, by making it, via Equation (18), parameter-dependent), the parameter-dependence becomes inherited also by the related Hilbert-space triplet. One of the most important consequences of the generalization

(19) $$\left\{L,K,H\right\}\;\to \;\left\{L_{\lambda },K_{\lambda },H_{\lambda }\right\}$$

is that the standard concept of the smallness of perturbations retains its intuitively acceptable physical meaning only in the “direct” flowchart scheme of Equation (10), i.e., only in the textbook Hilbert space L. The principle of correspondence enables us to quantify there what is a “small” and “large” perturbation term in (3). No such guiding principle survives the transition to the upper-case perturbed Hamiltonians.

The key point is that in the crypto-Hermitian picture even the very physical interpretation of the observable quantities becomes parameter-dependent (cf. Ref. [6] or Equation (19)). We know (see Section 3.2 or our recent comment [28]) that in the “inverted” 3HS flowchart (18) one encounters also the emergence of new free parameter(s) in metric. In other words, the ambiguity is enhanced. This implies the necessity of selection of a method of the removal of the formal ambiguity of the metric. The reconstruction of physics (i.e., in essence, a return to L) has simply the meaning of a return to the principle of correspondence.

### 4.2. A Merger of Two Hilbert-Space Triplets Requiring ${\;L}_{\lambda }=L$

The Dieudonné’s analysis of the possible formulation of quantum mechanics covering the general unbounded quasi-Hermitian Hamiltonians [21] did not find, due to its discouraging sceptical tone, too many applications in physics. The idea was only accepted and became popular after Bender with coauthors [4,7] proposed the restriction of the class of non-Hermitian Hamiltonians with real spectra to a “tractable” subset exhibiting PT symmetry (i.e., antilinear parity-times-time-reversal symmetry). With multiple technical details covered by reviews (e.g., [7,8]) and books (e.g., [9,33]), the key message was that the PT symmetry assumption leads to a thorough simplification of the technicalities.

In the more general setting, without a direct reference to PT symmetry, a thorough review of the eligible techniques of construction of the metric may be found in [8]. One has to admit that, even when a given Hamiltonian *H* with a real spectrum is successfully assigned a suitable “Hermitizing” metric Θ=Θ(H), the result is heavily ambiguous [28,31] plus, in any case, feasible just for the sufficiently elementary models (cf., e.g., [34,35]).

One is forced to expect that the difficulties will further grow if the initial Hamiltonian *H* gets perturbed. Even the simplifications resulting from the assumptions of PT symmetry may fail to apply when one tries to study the role and impact of perturbations. For example, a constructive disproof of applicability of the perturbation expansions of bound states using the conventional Rayleigh–Schrödinger power-series ansatzs using the *bona fide* “small” modification (18) of the Hamiltonian near an exceptional-point singularity was given in [36]. In our present paper, therefore, we will assume that the unperturbed Hamiltonian *H* is safely diagonalizable and lying sufficiently far from the influence of the possible exceptional-point-related pathologies.

Once we pick up some non-vanishing value of λ≠0 in the perturbed Hamiltonian, the most natural (and, in fact, the only properly interpreted) merger of this case with its unperturbed, exactly solvable λ=0 predecessor may be realized within the textbook Hilbert space L. In this sense, we may identify $L_{\lambda }$ with L. At this instant, our theoretical analysis of perturbations may start by a return to the unperturbed Hamiltonian *H* (defined in K) and to the related (and, presumably, also explicitly known) physical metric Θ in Hilbert space H. Subsequently, an additional, methodically motivated assumption of our knowledge of the Dyson-map factors Ω of the metric would open the way towards the reconstruction of the unperturbed physics in L. In the latter space, we may introduce the perturbations, purely formally, by Equation (3). This completes the merger of the unperturbed “inverted” 3HS diagram (12) with its Dyson-like “direct” 3HS perturbation-containing extension (10). The following, most natural five-Hilbert-space (5HS) perturbation-theory flowchart is obtained as a result.

(20) user—friendlyspace,exactsolvabilityinK,Hermiticitylost,H≠H†⟵(Θ→I)unperturbedsystemHermiticityofHinH:H=Θ−1H†Θ=H‡unperturbedmapΩ↙unperturbedh=ΩHΩ−1=h†inuser−unfriendlyspaceL(thetwospacescoincide)‖perturbedhλ=ΩλHλΩλ−1=hλ†definedinthesame_spaceLλ—dependentmapΩλ−1↘perturbedHamiltonianinλ—dependentspaceKλ,“tractable”Hλ⟶(Θλ=Ωλ†Ωλ)perturbedsystem,HermiticityinHλ:Hλ=Θλ−1Hλ†Θλ=Hλ‡.

Obviously, an immediate consequence of our decision of the identification of the two textbook Hilbert spaces L and $L_{\lambda }$ and of starting from the solvable unperturbed Hamiltonian *H* is that, in applications, the dynamical input information has an “inaccessible”, L—space-based form (3) of the lower-case operator v. We encounter the necessity of redirecting the 5HS flowchart of Equation (20).

## 5. Inverted 5HS Flowchart and Its Simplifications

In diagram (20), it is natural to require the full knowledge of the unperturbed system, i.e., of the upper case Hamiltonian *H* acting in K, of its metric Θ and factors Ω, and of the lower-case Hamiltonian h acting in L. As already indicated, the rest of the flowchart may be then read as an analogue of the “direct” pattern of Equation (10). Unfortunately, this only allows one to start from a lower-case perturbation term $\lambda \;v_{\lambda }$ in L, which is, due to the overall Dyson-inspired philosophy, prohibitively complicated. The simplifications, if any, can only be based on an intuitive insight helping us to pick up an appropriate *ad hoc* Dyson map $\Omega_{\lambda }$ leading to the expected thorough simplification of the new Hamiltonian $H_{\lambda }$.

In general, one can hardly rely upon the similar intuitive guidance, and one has to start perturbation expansions directly from the hypothetical dynamical input knowledge of the upper-case perturbation. In other words, at least some of the arrows in our 5HS flowchart (20) have to be inverted.

### 5.1. Formal Elimination of ${\;K}_{\lambda }$

In the 5HS recipe, the first, most visible challenge lies in the manifest λ-dependence of the working Hilbert space $K_{\lambda }$. A remedy consists of a transition from the λ-dependent space $K_{\lambda }$ to its λ-independent version K. The purpose can be served by an abstract operator $J_{\lambda }$ which would link the two spaces,

(21) $${|\psi \rangle }_{\lambda }\in K_{\lambda }\;\;\;\;\;|\psi \rangle =J_{\lambda }{|\psi \rangle }_{\lambda }\in K\;;$$

the perturbed Hamiltonian is then defined in $K_{\lambda }$ and reads

(22) $$H_{\lambda }=\Omega_{\lambda }^{-1}h_{\lambda }\Omega_{\lambda }\;.$$

Only now we should formally introduce a “tractable” upper-case operator *W* representing the input dynamical information about the perturbation,

(23) $${\left(J_{\lambda }^{†}\right)}^{-1}H_{\lambda }\;J_{\lambda }^{-1}=H+\lambda \;W_{\lambda }\;$$

because such a form of perturbation is defined in K. It should be treated as our initial information on dynamics. The 5HS flowchart (20) must be, in this sense, not only inverted but also re-read—as a perturbation-theory analogue of the 3HS reconstruction recipe prescribed by diagram (12).

Fully in the spirit of the specific Bender’s interpretation of the theory [7], also one of our present most fundamental methodical requirements is that the quantum system in question is not an open system and that its evolution is unitary. In L, the perturbation term must be self-adjoint, $v_{\lambda }=v_{\lambda }^{†}$. Otherwise, we could not accept any given candidate for the Hamiltonian, irrespectively of its particular representation, as an operator of an experimentally realizable observable. The emergence of *any* non-Hermiticity in $v_{\lambda }$ would indicate that our system ceased to be isolated from its environment. Being exposed to an external force, weak or strong, all of its measurable features could suffer a decay or growth.

Naturally, the unitarity restriction and the Hermiticity assumption are widely accepted when one works in the conventional framework of textbook space L. These requirements must be translated into the language of the sophisticated, λ-dependent physical Hilbert space $H_{\lambda }$. This being said, the calculations have to be performed in, and only in, Hilbert space K. Only the standard probabilistic interpretation of the results must be pulled down and performed, via metric $\Theta_{\lambda }$, in $H_{\lambda }$.

### 5.2. The Effective Physical Metric $\;T_{\lambda }\;$ in K Space

The initial, slightly counterintuitive motivation of the practical use of the Dyson-inspired 3HS constructions lied in the merits provided by the parallel representation of a single quantum state ψ in two different Hilbert spaces L and K. As long as they were assumed connected by a non-unitary map Ω, *both* the auxiliary space K and auxiliary Hamiltonian *H* become Ω-dependent in general. In the systems with perturbations, this observation led us to the necessity of working with the 5HS scheme (20) in general.

In preceding paragraph 5.1, the latter scheme has been complemented by the assumption of the existence (i.e., of our knowledge) of a further (trivial or nontrivial, unitary or non-unitary) auxiliary abstract mapping $J_{\lambda }:K_{\lambda }\to K$. This enabled us to replace the construction starting from the complicated input knowledge of $H_{\lambda }$ of Equation (22) by an alternative construction starting from another operator $H+\lambda W_{\lambda }$.

We now intend to show that, although the latter, new form of the dynamical input information is provided in another, *unperturbed* Hilbert space K (cf. Equation (23)), one need not necessarily return to $K_{\lambda }$ and reconstruct, ultimately, the correct physical Hilbert space $H_{\lambda }$. Indeed, the pattern can be shortened. A new, “effective” physical Hilbert space may be introduced for such a purpose (it is to be denoted as $M_{\lambda }$ in what follows).

In this spirit, the applied 3HS theory may be characterized, for unperturbed as well as perturbed systems, by the separation of descriptions of mathematics (in “friendly” $K_{\lambda }$) and of physics (in $H_{\lambda }$). Such a split of the two traditional roles of the single textbook Hilbert space L may simplify the picture. A deeper analysis of the change may be based on the factorization of the perturbed Dyson’s map into its unperturbed part and a correction which is, presumably, small,

(24) $$\Omega_{\lambda }=\Omega \;\left(1+\lambda \;\Delta_{\lambda }\right)J_{\lambda }\;.$$

Once we know the map between $K_{\lambda }$ and K, we may also factorize the “perturbed” metric accordingly,

(25) $$\Theta_{\lambda }=J_{\lambda }^{†}\;T_{\lambda }\;J_{\lambda }\;\;\;\;\;\;T_{\lambda }=\left(1+\lambda \;\Delta_{\lambda }^{†}\right)\;\Theta \;\left(1+\lambda \;\Delta_{\lambda }\right)\;.$$

This enables us to parallel the reconstruction step (B) of the general 3HS recipe (12). As long as we have $T_{\lambda }\neq \Theta_{\lambda }$ and $K_{\lambda }\neq K$, and as long as the metric depends on λ≠0, the reconstruction does not yield the original physical Hilbert space $H=H_{\lambda }$ of diagram (20) but rather another physical Hilbert space. This space would be an additional component of the theory. It deserves to be denoted by a separate dedicated symbol, say, $M_{\lambda }$. Thus, we arrive at the following operational interpretation of the scheme.

**Lemma** **2.** *After a transfer of the theory from $K_{\lambda }$ to K via Equations (22) and (23), the crypto-Hermiticity constraint (13) imposed upon the Hamiltonian may be given the form*

(26) $$\left(H^{†}+\lambda \;W^{†}\right)\;T_{\lambda }=T_{\lambda }\;\left(H+\lambda \;W\right)\;.$$

Given the perturbation λW, this equation specifies the (empty or non-empty) class of admissible (and, in general, non-unique) inner-product metrics $T_{\lambda }$ converting our working Hilbert space K into an innovated physical Hilbert space $M_{\lambda }$. Diagrammatically, we have

(27) perturbationprescribed,byHamiltonianH+λWλ,inλ—independentuser-friendly_spaceK(hermitization)⟶reducedmetricTλconvertsKintoanother,reducedphysical_spaceMλ(reducedmap)↘↙↗(thesamephysics)probabilisticinterpretationreconstructed,viaisospectralhλ=h0+λvλ=hλ†,inλ—independenttextbook_spaceL.

This represents the ultimate compact pattern suitable for the treatment of perturbations in quasi-Hermitian or PT-symmetric quantum theories. We have to conclude that, in these upgrades of the conventional formulation of quantum mechanics in the Schrödinger picture, the answer to the key question “Which perturbations are small?” is much less straightforward than one would a priori expect.

## 6. Perturbation Series in Crypto-Hermitian Physics

In our preceding analysis, we demonstrated that, in the theories using the non-self-adjoint representations of observables, a proper treatment of perturbations is far from trivial. We managed to simplify the general mathematical pattern, but its implementations still pose a few open problems.

### 6.1. Upper-Case Perturbations λW(λ) in K Space

One of the important tacit assumptions lying behind our preceding considerations is that the unperturbed Hamiltonian *H* is well-behaved and diagonalizable. Naturally, in an overall context of PT-symmetric quantum mechanics, such a property of *H* is fragile because, in general, such an operator may still exhibit a high degree of non-Hermiticity in K. In such a case, a word of warning was issued in our recent paper [13]. We studied a specific family of toy models there in which the Hamiltonians of interest were “almost equal” to a non-diagonalizable Jordan-block *N* by *N* matrix. In the paper, the model has been found “almost non-perturbative” simply because its Hamiltonians *H* were all lying very close to the Kato’s [1] exceptional-point dynamical extreme. For this reason, even the “unperturbed” metric Θ happened to be (almost) singular. This means that the model itself did not certainly satisfy the mathematical assumptions of smoothness and applicability of flowcharts (20) or (27).

Another, more physics-oriented word of warning was formulated in our brief comment [32]. We emphasized there that one of the key distinguishing features of the *closed* quasi-Hermitian quantum systems (i.e., e.g., of those exhibiting the spontaneously unbroken PT-symmetry [7]) is that the corresponding real manifolds of parameters may contain certain submanifolds (mostly, the hierarchies of hypersurfaces) which are formed by the associated Kato’s exceptional points. Naturally, these hypersurfaces determine the phase-transition boundaries of the (often, compact [37,38]) parametric domains of stability D of the quantum system in question. Again, we have to emphasize that, in any prospective analysis of stability, the applicability of our present constructive flowcharts only remains restricted to the “deep interiors” of D admitting the standard unitary-evolution interpretation of the closed quantum system in question.

Under these assumptions, we may expect that the pull-down process $K_{\lambda }\to K$ would not lead to any conceptual problems. We may merely require the knowledge of an appropriate input form (23) of the Hamiltonian. In the context of physics, we can also make use of the non-singular one-to-one correspondence between the textbook space L and its unitarily non-equivalent alternative $K_{\lambda }$. The existence of the invertible λ—dependent Dyson map (24) is then found equivalent to the stability of the quantum system in question.

**Lemma** **3.** *Whenever the spectrum of the perturbed Hamiltonian $H+\lambda \;W_{\lambda }$ (which is defined and non-Hermitian in the reduced, λ—independent Hilbert space K) is not real, the positive definite solution $T_{\lambda }=T_{\lambda }^{†}$ of Equation (24) does not exist.*

**Proof.**  As long as definition (25) implies the positive definiteness of metric $T_{\lambda }$ in $M_{\lambda }$ at any not too large λ, relation (26) should be re-read as a hidden Hermiticity requirement, i.e., as a spectral reality condition imposed upon our operator $H+\lambda \;W_{\lambda }$ in K. □

The perturbations-incorporating 5HS formalism offers an expected and consistent picture of correspondence between the loss of the reality of the spectrum (occurring at the Kato’s exceptional-point limit of $\lambda \to \lambda^{\left(EP\right)}$) and the loss of the unitary equivalence between the respective user-friendly and user-unfriendly physical Hilbert spaces $M_{\lambda }$ and L. In and only in this sense may we formally separate the class of perturbations $\lambda \;W_{\lambda }$ into its “sufficiently small” and “inadmissibly large” subclasses. At the same time, due to the manifest λ-dependence of $M_{\lambda }$, the formulation of some useful criteria of such a split would be much more difficult than in conventional quantum mechanics (a persuasive illustrative example may be found, e.g., in [13]).

### 6.2. Correspondence Principle and Lower-Case Perturbations λv(λ)

At every not too large value of λ the statement of Lemma 3 can be inverted in the sense that the reality of the non-degenerate perturbed spectrum provides access to the λ-dependent physical Hilbert space $M_{\lambda }$. However, this would be an observation of little practical value because we only know the unperturbed spectrum in advance. This is an obstacle which could invalidate the routine direct analysis of stability of given quantum systems with respect to random perturbations [32]. At the same time, in many quantum models, the “inaccessible” Hilbert space L still keeps carrying the role of a reliable interpretation background using the so-called principle of correspondence.

The applicability of such a principle to the closed quantum models defined directly in K appears, unfortunately, strongly limited. What remains at our disposal are only the possibilities of an indirect treatment of the correspondence and stability issues. Its realization could be based on a transfer of some time-proven requirements (e.g., of the norm-boundedness of the lower-case perturbations λv(λ)) from L to K. Naturally, nobody would ever use the complicated 3HS representation of unitary quantum systems if the conventional textbook space L were not practically inaccessible. Thus, the challenges emerging with the tests of stability appear directly connected to the inaccessibility of space L. A quantification of the smallness of the perturbation does not seem feasible without the necessary knowledge of the perturbed version of the metric.

The ultimate conclusion is that we are only allowed to use the K-space image of the inaccessible L-space perturbation,

(28) $$V_{\lambda }=\Omega^{-1}v_{\lambda }\;\Omega \;.$$

This enables us to recall the knowledge of the unperturbed matric Θ and the physical hidden-Hermiticity constraint

(29) $$V_{\lambda }^{†}\;\Theta =\Theta \;V_{\lambda }\;.$$

A relationship between operator $V_{\lambda }$ and the experimental information carried by the upper-case perturbation $W_{\lambda }$ of Equation (26) is still far from clear. As long as it is mediated by Equation (22) it may be given the form of a map from K to L,

(30) $$\left(h+\lambda v_{\lambda }\right)\Omega \left(1+\lambda \;\Delta_{\lambda }\right)=\Omega \left(1+\lambda \;\Delta_{\lambda }\right)\left(H+\lambda W_{\lambda }\right)\;$$

because after its minor re-arrangement, we obtain the full-fledged upper-case space—K version of correspondence between $W_{\lambda }$ and $V_{\lambda }$,

(31) $$\left(H+\lambda \;V_{\lambda }\right)\left(1+\lambda \;\Delta_{\lambda }\right)=\left(1+\lambda \;\Delta_{\lambda }\right)\left(H+\lambda \;W_{\lambda }\right)\;.$$

Such a relation confirms that $V_{\lambda }\neq W_{\lambda }$. A simplification of this formula can yield the explicit definition of the given, dynamical-input operator $W_{\lambda }$ in terms of $V_{\lambda }$ (with the norm-boundedness still under our control, in principle at least). Vice versa, the reconstruction of $V_{\lambda }$ from the given input $W_{\lambda }$ reads

(32) $$V_{\lambda }=\left(1+\lambda \;\Delta_{\lambda }\right)\;W_{\lambda }\;{\left(1+\lambda \;\Delta_{\lambda }\right)}^{-1}+\left(\Delta_{\lambda }\;H-H\;\Delta_{\lambda }\right)\;{\left(1+\lambda \;\Delta_{\lambda }\right)}^{-1}\;.$$

This was the last step towards the following important conclusion concerning the operational admissibility of the Hamiltonian.

**Lemma** **4.** *The insertion of operator $V_{\lambda }=V_{\lambda }\left(W_{\lambda }\right)$ of Equation (32) in Equation (29) yields the operator-equation criterion which tests the hidden Hermiticity of the perturbed Hamiltonian at any fixed component $\Delta_{\lambda }$ of the physical Hilbert-space metric $\Theta_{\lambda }$.*

In the scenario with constant input W≠W(λ), formula (32) implies a manifest λ-dependence of output V=V(λ) (or of v=v(λ)). The same observation would also hold in the opposite direction, moving from v≠v(λ) and V≠V(λ) to W=W(λ). In both of these scenarios, one may conclude that

(33) $$V_{\lambda }-W_{\lambda }=\Delta_{0}\;H-H\;\Delta_{0}+O\left(\lambda \right)\;.$$

This means that the difference between $V_{\lambda }$ and $W_{\lambda }$ (caused by the Hamiltonian-dependence of the metric) is, in general, model-dependent and non-perturbative, i.e., not necessarily small at small λ. In fact, such an observation exemplifies one of the differences between the present approach to (general, non-Hermitian) perturbations and the traditional textbook forms of perturbation theory.

### 6.3. Perturbation Expansions of Metric

The feasibility of the constructions at small λ is based on the analyticity assumptions and Taylor series

(34) $$W_{\lambda }=W_{0}+\lambda \;W^{\left(1\right)}+\lambda^{2}\;W^{\left(2\right)}+\ldots \;$$

(35) $$\Delta_{\lambda }=\Delta_{0}+\lambda \;\Delta^{\left(1\right)}+\lambda^{2}\;\Delta^{\left(2\right)}+\ldots \;.$$

Similarly, in Equation (25), we expand

(36) $$T_{\lambda }=\left[1+\lambda \;\Delta_{\lambda }^{†}\right]\;\Theta \;\left[1+\lambda \;\Delta_{\lambda }\right]=\Theta +\lambda \;T^{\left(1\right)}+\lambda^{2}\;T^{\left(2\right)}+\ldots \;$$

with

(37) $$T^{\left(1\right)}=\Delta_{0}^{†}\;\Theta +\Theta \;\Delta_{0}\;\;\;\;\;T^{\left(2\right)}={\left(\Delta^{\left(1\right)}\right)}^{†}\Theta +\Delta_{0}^{†}\;\Theta \;\Delta_{0}+\Theta \;\Delta^{\left(1\right)},$$

etc. These series may be inserted in the crypto-Hermiticity relation (26) of Lemma 2. In dominant order, the latter relation acquires the form that is satisfied by assumption,

$$H^{†}\Theta =\Theta \;H\;.$$

In the spirit of perturbation theory, the validity of this relation between the two given unperturbed operators *H* and Θ is granted. We may turn attention to the crypto-Hermiticity relation in the next-order approximation,

$$H^{†}\;T^{\left(1\right)}+W_{0}^{†}\;\Theta =\Theta \;W_{0}+T^{\left(1\right)}\;H\;.$$

It is to be interpreted as an equation by which one converts the known leading-order dynamical input (i.e., operator $W_{0}$) into the eligible output information about the first order correction $T^{\left(1\right)}$ to the (in general, non-unique) physical Hilbert-space metric. In the next step, one can proceed to the second order relation

$$H^{†}\;T^{\left(2\right)}+W_{0}^{†}\;T^{\left(1\right)}+{\left(W^{\left(1\right)}\right)}^{†}\;\Theta =\Theta \;\left(W^{\left(1\right)}\right)+T^{\left(1\right)}\;W_{0}+T^{\left(2\right)}\;H\;$$

which contains the operator $T^{\left(1\right)}$ obtained in the preceding step. The second-order correction $T^{\left(2\right)}$ to the metric is to be deduced. Along the same lines, one can also evaluate the higher-order corrections.

Alternatively, we might recall Equation (37) and redefine our task as the search for separate components of the Dyson map in expansion (35). In the first-order approximation, the reconstruction of $\Delta_{0}$ from the leading-order perturbation component $W_{0}$ has the form of equation

(38) $${\tilde{W_{0}}}^{†}\;\Theta =\Theta \;\tilde{W_{0}}\;\;\;\;\;\tilde{W_{0}}=W_{0}+\Delta_{0}\;H-H\;\Delta_{0}\;$$

in which we recognize relation (29). The abbreviation $\tilde{W_{0}}$ just represents perturbation $V_{\lambda }$ in the leading-order approximation of Equation (33). The variable λ is absent, and the interpretation of the equation is now approximative. Just the leading-order correction $\Delta_{0}$ is deduced from the leading-order input $W_{0}$.

## 7. Discussion

The Bender’s and Boettcher’s recommendation [4] of a turn of attention to complex potentials yielding real bound-state spectra inspired an increase of experimental as well as theoretical activities in several different (i.e., not necessarily just quantum) branches of physics [9,33,39]. In such a deeply innovative, fairly broad and flexible methodical framework, we tried to fill here one of the gaps in the theory by analyzing a slightly enigmatic problem of the response of a generic non-Hermitian system to perturbations. Let us now complement our mathematical results by a few physics-oriented remarks.

### 7.1. Physics of Open versus Closed Quantum Systems

During the study of quantum dynamics controlled by a parameter-dependent Hamiltonian $H_{\lambda }$ which happens to be non-Hermitian in a pre-selected and, in general, Hamiltonian-dependent Hilbert space $K_{\lambda }$, one has to distinguish between the open-system scenarios (reflecting, e.g., the decay of resonances) and the description of evolution of a closed system (characterized, first of all, by unitarity, i.e., by the conservation of norm). In our present paper, we paid attention to the latter dynamical regime. We were interested in a mathematically consistent description of quantum systems and of the changes of their observable properties (e.g., stability) under perturbations.

In a broader context of applied physics, the emergence of the concept of non-Hermitian Hamiltonians can, and should, be traced back to two independent sources. The older one was offered by Feshbach [40]. It consists of a reduction of the conventional Hilbert space of states L to a “model” subspace K. The trick helped to simplify the language of the theory in applications. In our present paper, only a marginal attention was paid to such a traditional non-Hermitian open-system theory. Interested readers may consult, e.g., monograph [41] for its recent review.

The basic idea of the study of the other, so-called closed quantum systems via non-Hermitian quantum Hamiltonians is usually attributed to Dyson [5]. In practice, the Feshbach- and Dyson-related model-building strategies based on non-Hermitian Hamiltonians appeared applicable in different situations. In contrast to the latter, 3HS strategy, its former, Feshbach-inspired counterpart deals with unstable systems and with their resonant states. Their evolution is, typically, controlled by a non-Hermitian effective Hamiltonian which may be even energy-dependent [42]. One might refer to the open-system model-subspace theory (in which the spectrum appears to be complex in general) under the acronym of “two-Hilbert-space (2HS) formulation of quantum mechanics”.

Occasionally, the 2HS–3HS parallels may be of interest. For example, space K must remain, from the user’s perspective, preferable to the textbook space L. In contrast to the 2HS case, the auxiliary 3HS space K must always be perceived as unphysical. An inner-product operator Θ of metric must be introduced to reconvert the unphysical space K into its correct alternative H.

### 7.2. The Problem of Stability

A not quite expected parallel emerges between the 1HS and 3HS criteria of admissibility and smallness of perturbations. In the conventional quantum theory, people usually expect that the stability of a system is lost whenever the perturbation λv(λ) ceases to be self-adjoint. Similarly, our present analysis came to the formally more complicated but analogous conclusion that, whenever we need to guarantee a closed-system stability (i.e., the reality of spectrum), the hidden Hermiticity constraint has to be imposed upon the perturbations λW(λ).

For a deeper understanding of the latter parallel, it is necessary to recall that, in the conventional Schrödinger representation, the unitarity of evolution is guaranteed by the self-adjointness of the Hamiltonian. The Dyson-inspired simplification of the Hamiltonian $h\to H=\Omega^{-1}\;h\;\Omega$ has been found useful even when accompanied by the loss of Hermiticity. Such an isospectral preconditioning is to be read as changing the Hilbert space into an auxiliary, unphysical one, L→K. A subsequent re-Hermitization of *H*, i.e., the introduction of a suitable inner-product metric operator Θ=Θ(H) then reconverts K into the third Hilbert space H which is physical again, i.e., unitarily equivalent to L. What is crucial is that any perturbation $H\to H_{\lambda }=H+\lambda \;W\left(\lambda \right)$ of the Hamiltonian in K necessarily induces the changes of geometry in the relevant physical Hilbert space, $H\to H_{\lambda }$. One must suspect that, after the inclusion of perturbations, the formalism might lose its internal mathematical consistency as well as any acceptable physical interpretation.

In our paper, the mathematical explanation of such an apparent paradox was described. Nevertheless, any exhaustive discussion of the physical aspects of the 5HS approach to the questions of stability must necessarily be model-dependent. Certainly, any guarantee of the smallness of perturbations must inseparably be related to the phenomenology-determining connection between the metric and a *complete* set $\left\{\Lambda_{j}\right\}$ of the non-Hermitian operators of observables, which is chosen as “irreducible in K” [27].

A more detailed analysis of this topic may be found in the physics-oriented review [6]. The ambition of the authors was to establish “a general criterion for a set of non-Hermitian operators to constitute a consistent quantum mechanical system”. One of the most successful implementations of this criterion was discovered and made popular by Bender with coauthors who studied multiple specific ordinary differential crypto-Hermitian quantum models exhibiting additional antilinear symmetries of $H=-d^{2}/dx^{2}+V\left(x\right)$ called PT—symmetry and PCT—symmetry (see, e.g., [7,8] for details).

### 7.3. The Requirement of Unbroken PT-Symmetry

The abstract 3HS formulation of quantum mechanics may be traced back to Dieudonné [21]. He pointed out, as early as 1961, that many of the rigorous mathematical aspects of the 3HS theory are rather discouraging in the general case. Later, a number of further critical arguments has been found against the use of unbounded Hamiltonians *H* (cf., e.g., [15]). A partial remedy has only been found in review [6] dated 1992. There, the authors circumvented some of the most serious formal drawbacks of the general 3HS formalism by admitting only such “quasi-Hermitian” observables (including Hamiltonians *H*) which are bounded in K.

A return of physicists to unbounded non-Hermitian Hamiltonians with real spectra appeared motivated, a few years later, by the needs of quantum field theory [43]. This inspired an intensive search for suitable toy models. In particular, for one-dimensional Schrödinger operators [4,44,45,46], a serendipitous benefit appeared to emerge from an exceptionally straightforward realization of the Hermitization K→H [7]. The goal has been achieved thanks to a fortunate combination of the PT-symmetry assumption

(39) HPT=PTH

(where P is parity and T represents the time reversal) with the postulate of the existence of an ad hoc operator C exhibiting properties of a charge [7]. This facilitated the construction of the metric because the Dieudonné’s relations (13) appeared satisfied by the product $\Theta_{\mathrm{PT}}=\mathrm{PC}$ [7,33]. Thus, in a widely accepted terminology referring to the original motivating context of quantum field theory, people usually connect the EP-related instant of the loss of the reality of the spectrum of *H* with the loss of the PT-symmetry of the wave functions, giving the loss the name of the “spontaneous breakdown of PT-symmetry” [7].

After 2012, several concrete implementations of the model-building strategy based on the antilinear symmetries (39) revealed that the rigorous mathematical background of such an approach may happen to be perceivably more complicated than originally expected (cf., e.g., [23,47,48,49,50]).

### 7.4. Physical Meaning of the Reality of the Energies

In the majority of the traditional textbooks on quantum mechanics [2], the exposition of the theory usually starts from the description of the closed systems, i.e., from the conventional bound-state Schrödinger equation which is, say, real-parameter-dependent. The evolution of these systems in time is unitary and, in the light of Stone theorem [3], the Hamiltonian itself is self-adjoint in the corresponding Hilbert space of states L.

Whenever people turn attention to the so-called open quantum systems, the self-adjointness constraint must be omitted because the spectrum of the energies need not be real anymore. Still, along strictly the same lines, one can also consider a number of more general, non-Hermitian resonant-state Schrödinger equations

(40) $$\hat{H(\lambda )}\;|\psi \left(\lambda \right)\rangle =\hat{E(\lambda )}\;|\psi \left(\lambda \right)\rangle \;$$

with non-real spectra $\left\{\hat{E(\lambda )}\right\}$. The search for the solutions need not be followed by any reconstruction of the metric because the topological vector space K of the open-system states |ψ(λ)〉∈K becomes physical [51].

The formal parallels between the closed and open quantum systems become even stronger when one is allowed to assume that the λ-dependence of the solutions remains weak. Then, one usually treats the Hamiltonian as composed of a reference operator and a small perturbation,

(41) $$h_{\lambda }=h+\lambda \;v=h_{\lambda }^{†}\;\;\;\;\;\;\hat{H_{\lambda }}=\hat{H}+\lambda \;\hat{V}\;\neq \;{\hat{H_{\lambda }}}^{†}\;.$$

It is not too difficult to proceed in a consequent parallel with the self-adjoint case. With obvious modifications: the conventional unperturbed bases must be replaced by their biorthonormalized generalizations. As long as $\hat{H_{\lambda }}\neq {\hat{H_{\lambda }}}^{†}$, one has to consider Schrödinger equations for the respective left and right eigenvectors of the Hamiltonian,

(42) $$\left[\hat{H_{0}}+\lambda \;\hat{V}\right]\;|\psi \left(\lambda \right)\rangle =\hat{E}\left(\lambda \right)\;|\psi \left(\lambda \right),\rangle \;\;\;\;\;|\psi \left(\lambda \right)\rangle \;\in \;K_{\lambda }\;$$

(43) $$\left[{\hat{H_{0}}}^{†}+\lambda \;{\hat{V}}^{†}\right]\;|\psi \left(\lambda \right)\rangle \;\rangle ={\hat{E}}^{*}\left(\lambda \right)\;|\psi \left(\lambda \right)\rangle \;\rangle \;\;\;\;\;|\psi \left(\lambda \right)\rangle \;\rangle \;\in \;K_{\lambda }\;.$$

The energies need not be real in general (so that the superscripted star ${}^{*}$ marks here the complex conjugation) but, under suitable mathematical assumptions, they can still be sought via the same Rayleigh–Schrödinger ansatz [1],

(44) $$\hat{E}\left(\lambda \right)=\hat{E}\left(0\right)+\lambda \;\hat{E_{1}}+\lambda^{2}\;\hat{E_{2}}+\ldots \;.$$

One of the key distinctive features of the hatted energies (44) is that they are, in general, complex. For this reason, as we already emphasized, we were only interested here in the “unhatted” Hamiltonians $H_{\lambda }$ possessing the strictly real spectra and exhibiting the additional hidden-Hermiticity property (7).

### 7.5. The Concept of Admissible Perturbations

On the basis of physical-reality-reflecting requirements, one always has to specify clearly the class of the experimentally realizable perturbations, irrespective of the formalism. Such a specification seems missing in the literature on 3HS models, so, in our recent comment [32] and paper [36], we turned attention to the topic. We pointed out that the variations of the Hamiltonian cause also the variations of the geometry of the underlying physical Hilbert space. We found the consequences “strongly counterintuitive”. In the present continuation of these considerations, we offered one of the possible resolutions of the puzzle, reflecting the well-known danger [52] that the presence of exceptional points at small λ could endanger the convergence of Rayleigh–Schrödinger series. For this reason, the acceptability of our present approach is based on the assumption that these singularities stay sufficiently remote so that the consequent perturbation-approximation strategy remains admissible and mathematically well founded.

Let us point out that the Hilbert-space geometry (controlled by the physical inner-product metric Θ) can acquire its λ-dependence in two complementary ways, viz, directly and indirectly. Besides the obvious, “direct” λ-dependence inherited from the Hamiltonian, one must take into account that the assignment H→Θ(H) remains, at every λ, ambiguous. Thus, indirectly, the removal of this ambiguity also remains at our disposal as a phenomenologically relevant independent degree of freedom in model-building [6,28]. The latter observation was a core of our present construction of the general re-Hermitization scheme admitting the analysis of the admissibility of the perturbations.

In diagram (20), we showed, in particular, that it is necessary to distinguish between as many as five relevant Hilbert spaces, in principle at least. In practice, the pattern admits various simplifications. We succeeded in reducing the set of the relevant spaces to three (cf. diagram (27)). This renders the explicit perturbation–expansion constructions of physical states possible. These constructions are expected to be based again on the most natural power-series ansatzs sampled by Equation (44). Thus, our technique of description of the perturbed unitary quantum systems does not seem to lead to any inconsistencies.

### 7.6. Outlook

Any realistic application of our perturbation-expansion recipe will be strongly model-dependent so that it remained beyond the scope of our present paper. We only paid detailed attention to a few key technicalities. Under the assumption that the λ-dependence of our Hamiltonians $H_{\lambda }$ happens to be analytic, we managed to show that the extension of the conventional perturbation-expansion techniques to the PT-symmetry context is, up to a few important differences, straightforward. One of the main explicit technical messages delivered by our present paper is that, whenever the unitary, 3HS-represented quantum system with the Hamiltonian *H* which is non-self-adjoint in K but self-adjoint in H gets exposed to a perturbation λW which is non-self-adjoint in K, there exists an appropriate generalization of the textbook perturbation theory which may be constructed in K and which gives the correct physical predictions.

We clarified that the traditional notions of the norm or of the smallness of perturbations in L cannot be too easily transferred to the 3HS framework. Whenever the conventional perturbed Hamiltonians $h_{\lambda }=h+\lambda v_{\lambda }$ are not found prohibitively complicated, the analysis of perturbations should certainly be performed in the textbook space L. Only when this is not the case does a transition to the quasi-Hermitian picture become a viable strategy. Indeed, even though the non-Hermitian representation approach proves reasonably straightforward and mathematically well founded, it still remains not too easy to implement.

Our key message concerns the criteria of the smallness of perturbations. In contrast to the conventional Hermitian perturbation theories [2], and in contrast to the studies of the response to perturbations in open quantum systems [15], a *decisive* component of any mathematically consistent perturbation-approximation description of any closed crypto-Hermitian model lies in the reconstruction of the physical Hilbert-space metric $\Theta_{\lambda }$. One encounters here a hidden parallel with the Hermitian theories: The “operationally admissible” perturbations λW(λ) are only those which are self-adjoint in the physical Hilbert space. As long as the physical norm (i.e., the metric) necessarily varies with the parameter λ in general, one cannot confirm or disprove the smallness of a given quasi-Hermitian perturbation λW(λ) too easily (interested readers might find a fairly discouraging illustrative example in [13]).

For practical purposes, this implies that, as long as the construction of $\Theta_{\lambda }$ remains an inseparable part of the 5HS formalism, the questions of stability of a given system with respect to random perturbations (answered, in open systems, by the construction of the pseudospectrum [15,18]) do not seem to have any easy or exhaustive answer for the closed crypto-Hermitian quantum systems at present. Mostly, only an a posteriori, self-consistent test of the smallness of perturbation remains at our disposal.

A certain consolation may only be sought in the fact that this is, after all, a situation which is not too different from the situation in the single-Hamiltonian setting. Indeed, the 5HS ambiguity of the specification of the “admissible” physical λ≠0 Hilbert space $H_{\lambda }$ is merely inherited from the unperturbed 3HS formalism, where λ=0. It can always be suppressed, in principle at least, via an extended dynamical input access to a complete set $\left\{\Lambda_{j}\right\}$ of the non-Hermitian operators of observables in K. Fortunately, citing the words of review [6], “it is not always necessary to construct the metric for the whole set of observables under consideration” in applications.

## 8. Conclusions

In multiple applications, the Rayleigh–Schrödinger perturbation series is found convergent. According to the Kato’s book [1], the radius of the corresponding circle of convergence $\lambda_{\max}$ is equal to the distance of λ=0 from the (in general, complex) set of the so-called exceptional-point (EP) values $\lambda^{\left(EP\right)}$. In this way, the question about the “smallness” of perturbations λv is given a more or less exhaustive abstract answer. Unfortunately, this answer is not too constructive even in Hermitian theory where the EP values are never real. Thus, people work with the analytically continued Hamiltonians even though they cease to be self-adjoint.

Our present study of crypto-Hermitian $H_{\lambda }$s may be summarized as reopening the problem of the smallness of perturbations. Our starting point was the standard 3HS theory, the probabilistic interpretation of which is known to be obtained via a positive definite, Hamiltonian-dependent physical Hilbert-space metric. We observed that, once one admits the λ-parametric variability of the Hamiltonian $H_{\lambda }$, the task of making the physical contents of the theory consistent becomes nontrivial even when the variability of $H_{\lambda }$ is only caused by a “small” perturbation in $H_{\lambda }=H+\lambda W$ (in the unperturbed limit λ→0, we dropped the usual zero subscript as redundant).

A key theoretical obstacle was found in the transfer of the parameter-dependence from $H_{\lambda }$ to all of the components of the formalism and, in particular, to the Dyson’s map ($\Omega \to \Omega_{\lambda }$). At length, we discussed the resulting parameter-dependence modifications of the mathematical manipulation space ($K\to K_{\lambda }$) as well as of the ultimate physics-representing space ($H\to H_{\lambda }$).

We had to apply the 3HS formalism twice, viz., to the unperturbed Hamiltonian *H* and, separately, to its perturbed partner $H_{\lambda }$ associated with the modified, λ-subscripted Hilbert-space triplet $\left\{L_{\lambda },K_{\lambda },H_{\lambda }\right\}$. Without any loss of generality, we postulated the λ-independence of the textbook Hilbert spaces ($L_{\lambda }=L$). This enabled us to merge the two respective Dyson-inspired 3HS formulations of quantum mechanics of Ref. [6] into a single 5HS flowchart.

In the resulting constructive recipe, a number of subtle technical as well as physical interpretation problems emerged (one of them, with $\lambda_{\max}=0$, was recently reported in [13]). Just a smooth jump from λ=0 to λ≠0 was therefore considered. Some of the consequences were discussed. It may be concluded that the 5HS formulation of perturbation theory is nontrivial but still feasible and reasonably transparent and user-friendly.
